# New Antibiotics for Multidrug-Resistant Bacterial Strains: Latest Research Developments and Future Perspectives

**DOI:** 10.3390/molecules26092671

**Published:** 2021-05-02

**Authors:** Marco Terreni, Marina Taccani, Massimo Pregnolato

**Affiliations:** Department of Drug Science, University of Pavia, Viale Taramelli 12, 27100 Pavia, Italy; marco.terreni@unipv.it (M.T.); marina.taccani01@universitadipavia.it (M.T.)

**Keywords:** antibiotic resistance, MDR bacterial strains, new antibiotics

## Abstract

The present work aims to examine the worrying problem of antibiotic resistance and the emergence of multidrug-resistant bacterial strains, which have now become really common in hospitals and risk hindering the global control of infectious diseases. After a careful examination of these phenomena and multiple mechanisms that make certain bacteria resistant to specific antibiotics that were originally effective in the treatment of infections caused by the same pathogens, possible strategies to stem antibiotic resistance are analyzed. This paper, therefore, focuses on the most promising new chemical compounds in the current pipeline active against multidrug-resistant organisms that are innovative compared to traditional antibiotics: Firstly, the main antibacterial agents in clinical development (Phase III) from 2017 to 2020 are listed (with special attention on the treatment of infections caused by the pathogens *Neisseria gonorrhoeae*, including multidrug-resistant isolates, and *Clostridium difficile*), and then the paper moves on to the new agents of pharmacological interest that have been approved during the same period. They include tetracycline derivatives (eravacycline), fourth generation fluoroquinolones (delafloxacin), new combinations between one β-lactam and one β-lactamase inhibitor (meropenem and vaborbactam), siderophore cephalosporins (cefiderocol), new aminoglycosides (plazomicin), and agents in development for treating drug-resistant TB (pretomanid). It concludes with the advantages that can result from the use of these compounds, also mentioning other approaches, still poorly developed, for combating antibiotic resistance: Nanoparticles delivery systems for antibiotics.

## 1. Introduction

Discoverer of penicillin Alexander Fleming, in December 1945, during his acceptance speech of the Nobel Prize in Medicine, announced the risk of the inevitable phenomenon of antibiotic resistance, already observed in laboratories, with the following words:

“*it’s not difficult to make microbes resistant to penicillin in the laboratory by exposing them to concentrations not sufficient to kill them…there is the danger that the ignorant man may easily under-dose himself and, by exposing his microbes to non-lethal quantities of the drug, make them resistant*.”

(A. Fleming, Penicillin, Nobel Lecture, 11 December 1945)

Fleming’s predictions turned out to be accurate: The incorrect use, sometimes real abuse, of antibiotics, speeds up the development and spread of bacteria resistant to them.

Considering the penicillin as an example, if bacteria are subjected to “non-lethal levels” of the antibiotic, they can use it as a signaling with regulatory functions. Bacteria can release β-lactamase enzymes, that hydrolyze the amide bond of the four-membered β-lactam ring resulting in the inactivation of the β-lactam antibiotic.

The reported case is just one of the many defense mechanisms that bacteria have against antibiotics. Antibiotics have undoubtedly been a milestone in the history of humanity and modern medicine; they are an indispensable and life-saving weapon against numerous infectious diseases, including the ones associated with organ transplants, cancer chemotherapies, and intensive therapies. In the last century, research has produced many new antibiotics; however, since the 1990s, the number of antimicrobial agents discovered has been in sharp decline, with a simultaneous and worrying increase in the phenomenon of antibiotic resistance. Bacteria showing resistance to at least three different classes of antimicrobials, defined as multidrug resistant (MDR), have become common, especially in hospitals; there is a risk of entering a so-called “post-antibiotic era” in a few years, in which infections apparently under control easily turn into lethal threats. It is evident to everyone that antibiotic resistance is one of the main health problems nowadays, with a strong impact both clinically and economically. Pathogens such as methicillin-resistant *Staphilococcus aureus* (MRSA) and vancomycin-resistant *enterococci* (VRE) have become extremely difficult to eradicate. It is estimated that each year, more than 2.8 million people in the United States alone contract an infection resistant to traditional antibiotics, causing more than 35,000 deaths [1]. In Europe, antibiotic resistance is responsible for about 33,000 deaths per year [2]. Globally, pneumonia and blood infections that cause sepsis contribute heavily to infant mortality in the first five years of life. Approximately 30% of newborns with sepsis die from bacterial infections resistant to traditional antibiotics [3]. In 2016, the World Health Organization (WHO) published a list of the world’s leading antibiotic-resistant bacteria, for which there is an urgent demand for new treatments [4]. The aim is certainly to help countries accelerate national surveillance, control, and research activities for new active ingredients. The list is divided into three categories, each describing the risk associated with the antibiotic-resistant bacterial species: Critical, high, and medium. *Mycobacterium* (including *M. tuberculosis*, responsible for 1.8 million deaths per year worldwide) has not been included in this list because it is a long-established threat. Note that Gram-negative bacteria pose a looming danger.

Pathogens of the genera *Acinetobacter*, *Pseudomonas*, and *Enterobacteriaceae* (including *Klebsiella pneumoniae*, *Escherichia coli*, *Enterobacter* spp., *Serratia* spp., *Proteus* spp., *Providencia* spp., and *Morganella* spp.) are the most feared in hospitals, nursing homes, and aged care facilities, where related infections can be lethal. These pathogens have already developed resistance to carbapenems, which are extremely powerful antibiotics, often used as life-saving drugs in hospitalized patients. In fact, the rapid increase in the number of infections caused by carbapenem-resistant *Enterobacteriaceae* (CRE), which produce carbapenemases (especially *K. pneumoniae* carbapenemase) capable of hydrolyzing and inactivating carbapenems and β-lactams, is alarming. Of high priority are VRE, MRSA, *Helicobacter pylori* (the first risk factor for stomach cancer), *Campylobacter* spp. (responsible for the highest number of food contaminations in Europe), *Salmonella* spp. (food poisoning), and *Neisseria gonorrhoeae* (causes gonorrhea, a sexually transmitted disease). Finally, *Streptococcus pneumoniae* (responsible for the majority of community-acquired pneumonia), *Haemophilus influenzae* (related to respiratory infections), and *Shigella* spp. (transmitted through water or foods contaminated with feces, causes dysentery) are placed in the category of medium priority.

In addition, it should be pointed out that the potential spread of resistant organisms also has a negative impact on the health of subjects not directly exposed to certain antibiotics. Faced with this scenario, which is no longer science fiction but a reality, there is an urgent need for new antibacterial active ingredients in order to ensure effective treatments against infections resistant to traditional antibiotics.

In recent years, awareness of the issue of antibiotic resistance has increased, including in the political field: In 2017, the G20 countries decided to intensify global collaboration on this issue to stimulate the R&D of antimicrobial molecules, also starting from existing antibiotics. Since 2017, eight new antibiotics have been approved by the FDA, including one for the treatment of multidrug-resistant tuberculosis: Most of these drugs were developed from traditional molecules and target *Enterobacteriaceae* resistant to carbapenems and other pathogens considered dangerous by WHO [4].

The following paper examines from a chemical and clinical point of view the most promising new compounds still under preclinical and clinical investigation, including the active ingredients that entered Phase III last year and the agents of pharmacological interest that gained market authorization between 2017 and 2020. The latest results of the research are shown together with the strategies necessary to stem the problem of antibiotic resistance, concluding with perspectives for the future.

## 2. Preventive Strategies and Measures to Curb Antibiotic Resistance

Antibiotic resistance threatens modern medicine, and above all the effectiveness of a decisive and prompt global health response to infectious diseases, due to systematic abuse and excessive use of antibiotics in human medicine and food production. Indeed, the massive or inappropriate use of such drugs in humans, animals or agriculture results in the emergence of drug-resistant microorganisms evolved under this strong selective pressure. In 2015, aware of the huge problem of antibiotic resistance, the WHO decided to adopt the Global Action Plan on Antimicrobial Resistance, based on five strict objectives: To improve awareness and understanding of antimicrobial resistance; to strengthen knowledge and the amount of data; to reduce the incidence of infections through effective hygiene measures; to optimize the use of antimicrobial drugs in human and animal health; and to increase investment in new drugs, diagnostic tools, vaccines, and other interventions [5]. In addition to the WHO, there are other associations such as the Food and Agriculture Organization of the United Nations and the World Organization for Animal Health that give ample space to the fight against antibiotic resistance. The use of antibiotics in veterinary medicine is extremely important: It is necessary to strengthen the regulatory system for medicated food and feed, mainly used in intensive farming, in order to prevent the onset of infections due to the large number of animals raised in situations of confinement. To this end, the surveillance and monitoring systems for resistant bacteria and the indiscriminate use of antibiotics have multiplied, not only in human medicine, but also in veterinary. In general, it is good practice to avoid the repeated use of the same molecule and to increase patient compliance with correct drug dosages and timing. Regarding this, in 2013, the European Centre for Disease Prevention and Control (ECDC) published a paper reviewing procedures and guidelines to improve the compliance of health professionals with regard to the timing, dosage, and duration of peri-operative antibiotic prophylaxis for the prevention of infections in surgical rooms [6]. New molecules are therefore vital to overcoming the resistances that have developed as well as the need to empower the use of existing antibiotics and to promote the study of increasingly valid diagnostic tests for the identification of resistant bacteria and for determining antibiotic sensitivity.

## 3. Molecular Mechanisms of Antibiotic Inactivation

Bacteria are able to inactivate antibiotics through numerous molecular mechanisms [7]:

(a) Production of inactivating enzymes: The antibiotic loses its biological activity as it is precisely inactivated by specific enzymes produced by the bacterium. This happens, for example, in the case of β-lactam antibiotics that are hydrolyzed by β-lactamases. *Enterobacter* spp. produce extended-spectrum β-lactamases (ESBL) with the same inactivating function, becoming difficult to eradicate. Other enzymes capable of inactivating certain antibiotics are acetyltransferase, phosphotransferase, and adenyltransferase.
(b) Changes and alterations in the antibiotic target: This happens, for example, in resistance to erythromycin, wherein the methylation of an adenine residue in the peptidyl-transferase of r-RNA 23S decreases its affinity for the antibiotic without damaging protein synthesis. Another important case is the modification of penicillin binding proteins (PBPs) by MRSA.
(c) Reduced cellular permeability: The penetration of an antibiotic can be reduced by structural changes in the cell’s surface casings. In Gram-negatives, the resistance may be due to an alteration or quantitative decrease in porines, or proteins through which many antibiotics penetrate. They delay the incoming flow of numerous antibiotics thanks to different mechanisms that include limitation in relation to molecular size, hydrophobicity, and charge repulsion, thus contributing to the intrinsic resistance of many microorganisms. This is the case for *Pseudomonas aeruginosa*, which shows resistance to imipenem.
(d) Increased outflow: Antibiotics taken-up into bacteria cell are removed by energy-driven drug efflux systems. Activation of alternative metabolic pathways: The case of sulfamidics is explanatory. Bacteria treated with sulfamidics, in fact, still manage to synthesize folic acid through alternative metabolic pathways.

## 4. Main Agents in Clinical Development (Phase III) in 2020

Currently, the search for new antimicrobial active ingredients is largely led by small- and medium-sized enterprises, as large pharmaceutical companies continue to abandon such projects. In fact, the return on investment for antibiotics that have been marketed in recent decades has been rather negative. In 2011, an analysis entitled “Challenges of Antibacterial Discovery”, published in the journal *American Society for Microbiology*, referred to a “discovery void” that had persisted since 1987 without significant patents or advances [8]. Unfortunately, it is difficult to obtain incentives to develop and study new antibiotics, for various reasons. Many of the molecules selected in the laboratory that are directed against enzyme targets quickly end up developing resistance in the pathogens examined. An active substance is considered innovative if it does not show the phenomenon of cross-resistance to existing antibiotics. In this context, cross-resistance is defined as resistance within the same class of antibiotics, which can be quantified by systematic, in vitro susceptibility tests to genetically determined pathogens. If sufficient information on cross-resistance is absent or not available, an active substance is considered innovative if it belongs to a new class of antibiotics (new scaffold or pharmacophore), if it has a new target or binding site that has never been present before, or if it shows a new mechanism of action.

Antibiotics discovered in the so-called “golden age” of the last century were generally very complex natural products with numerous molecular targets, so the phenomenon of resistance was less common during clinical trials. The dosages of the most widely used antibiotics are usually in the range of hundreds of milligrams per day, so it is necessary that these active ingredients be extremely selective for bacterial targets in order to avoid toxic effects for the patient. However, some newly discovered molecules, active against multidrug-resistant bacteria, are not selective and can consequently cause important side effects. For example, three antibiotics, telithromycin, temafloxacin, and trovofloxacin, which were approved in the 1990s and early 2000s, were removed from the market due to serious adverse reactions, as they were not selective [9]. In addition, new antibiotics are usually administered in the hospital as a “last resort” in patients with complicated and multidrug-resistant infections not treatable with traditional antibiotics. It is therefore not difficult to understand why pharmaceutical companies prefer to avoid investing in the development of new antibiotics.

However, there are small start-ups that propose new approaches and a greater commitment to R&D. In addition, research involving public investments aimed at the development of new antibiotics has increased in recent years: The Global Antibiotic Research and Development Partnership was created in 2016. Aware of the need to ensure the availability of antibiotics even for patients undergoing chemotherapy or organ transplants, many countries around the world are implementing different initiatives to stimulate the research of innovative antibiotics. The new findings are not powerful enough weapons to combat the current challenges of antibiotic resistance, but it can be interesting to discuss the latest developments and highlight the compounds that appear most significant according to clinical studies. The current framework for pharmaceutical research and the development of new antimicrobial drugs is outlined by two 2020 reports: “Antibacterial agents in clinical development: An analysis of the antibacterial clinical development pipeline” [10] and “Antibacterial agents in preclinical development” [11], both compiled by the WHO’s Antibacterial Resistance Division.

Eight new antibacterial active ingredients, including one for the treatment of tuberculosis, have been approved since 2017. Pretomanid, an agent against multidrug-resistant tuberculosis, was developed by the non-profit organization TB Alliance. About half of the newly approved antibiotics target the carbapenem-resistant *Enterobacteriaceae* (CRE), oxacillinase-48-producing *Enterobacteriaceae* (OXA-48), and β-lactamase-producing *Enterobacteriaceae* (ESBL). Sixty products are in clinical development (as of 2020), including ten biological drugs. Among these different products under evaluation, 32 antibiotics are active against the most dangerous pathogens included in the WHO’s 2016 list (WHO priority pathogens), and many of them consist of combinations of new β-lactams and β-lactam inhibitors. Twelve antibiotics in clinical development target at least one of the critical Gram-negative pathogens. Antibiotics are still unable to treat carbapenem-resistant *Acinetobacter baumannii* and *P. aeruginosa*, even though the research on agents against tuberculosis and *Clostridium difficile* has made considerable progress [10].

Since 2019, the inhalation formulation of murepavadin (a polypeptide antibiotic), whose clinical trial regarding the intravenous formulation had been discontinued due to suspected nephrotoxicity, has been under development [10]. Murepavadin is the only potential treatment against Gram-negative bacteria that can meet all of the criteria of innovation, including the absence of cross-resistance within the same class of antibiotics.

However, if a compound does not meet all the criteria of innovation, it does not necessarily mean that it lacks therapeutic utility for particular categories of patients. Since the 2018 update, many new compounds have entered Phase I of clinical development. The two new oral inhibitors of topoisomerase (zoliflodacin and gepotidacin) have successfully passed Phase II clinical trials, entering Phase III. Lefamulin (new pleuromotilin) and the combination relebactam/imipenem/cilastatin have been approved by the FDA. In addition, worth mentioning is the approval of cefiderocol, a β-lactam antibiotic active against the three critical priority pathogens, by the FDA for complicated urinary tract infections. The largest proportion of Phase III antibiotics come from existing classes, especially β-lactams, fluoroquinolones, macrolides, oxazolidinones, and topoisomerase inhibitors. The most promising compounds are examined below.

### 4.1. Zoliflodacin in the Treatment of Multidrug-Resistant N. gonorrhoeae

The bacterium *N. gonorrhoeae*, resistant to third-generation cephalosporins and fluoroquinolones, is included in the category of high priority pathogens: There is an urgent need for new antibiotics that can overcome such resistance. *N. gonorrhoeae* is the causative agent of gonorrhea, a sexually transmitted disease that in women can remain asymptomatic for long periods but which is responsible, in severe cases, for severe complications such as infertility, ectopic pregnancies, and neonatal blindness.

In recent years, infections with *N. gonorrhoeae* resistant to penicillin and cephalosporins such as cefixime and ceftriaxone (usually used as the last therapy available in combination with azithromycin) have increased disproportionately. A recent report from the CDC—Centers for Disease Control and Prevention—documented over 500,000 new cases of gonorrhea in the United States during 2018 [12].

Since 2019, the compound zoliflodacin is in Phase III for the treatment of multidrug-resistant *N. gonorrhoeae*, developed by Entasis Therapeutics in collaboration with the Global Antibiotic Research Development Program [13]. It is the first synthesized antibiotic belonging to the class of spiropyrimidinetrions. It has a unique mechanism of action: It inhibits type II bacterial topoisomerase by binding to a different site than that of fluoroquinolones. The minimal inhibitory concentration (MIC) value together with pharmacokinetic parameters are regarded to have the greatest importance in the optimization of targeted antibiotic therapy [14]. The MIC50 provides the so-called “intrinsic activity” of an antimicrobial, while the MIC90, which is calculated on the basis of larger, inter-center studies, is a reflection of different resistance mechanisms of the species under investigation. Zoliflodacin shows a very low resistance frequency and is active not only against multidrug-resistant *N. gonorrhoeae* with a MIC between 0.002 and 0.25 μg/mL but also against some troublesome Gram-positive and Gram-negative bacteria.

**Figure 1:**
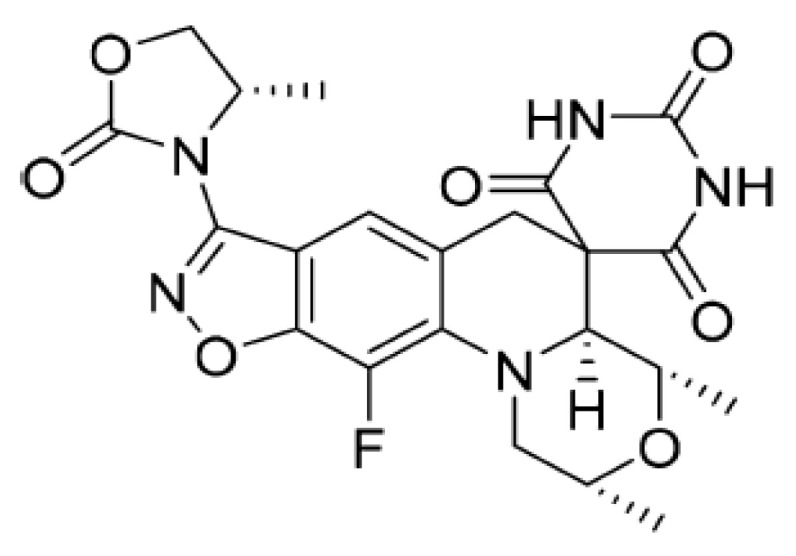
Formula of zoliflodacin.

From a chemical point of view, the formula of zoliflodacin (Figure 1) is based on a new benzisoxazole scaffold and contains the pyrimidinetrione spirocyclic pharmacophore, which gives its name to this innovative class of antibiotics. The structure activity relationship (SAR) of this molecule was developed using in vitro tests showing the mechanism of inhibition of DNA gyrase and antibacterial activity.

Several compounds in the benzisoxazole series have good activity against quinolone-resistant pathogens, including *S. aureus*, *S. pneumoniae*, and *H. influenzae*. The insertion of a substituent (4-methyl-1,3-oxazolidin-2-one) in position 3 of the benzisoxazole ring, provides derivatives with excellent antibacterial activity and better pharmacokinetic profile, an example is zoliflodacin, the most promising in the series of spiropyrimidinetriones.

Topoisomerase DNA are enzymes that control the three-dimensional conformation of DNA. Topoisomerases I and II are distinguished on the basis of their ability to cause single- or double-chain ruptures in DNA. DNA gyrase and topoisomerase IV are the two type II topoisomerases present in bacteria. Their different roles are fundamental in DNA replication. These enzymes are the target of the fluoroquinolone class.

DNA gyrase is composed of two subunits, GyrA (97 kDa) and GyrB (90 kDa); the active form being an A2B2 heterotetramer able to introduce negative supercoils into the DNA molecules. This process of supercoiling is crucial to allow DNA to re-enter newly created cells. Zoliflodacin, as ciprofloxacin (fluoroquinolone antibiotic), has the ability to inhibit bacterial topoisomerases much more selectively than mammalian topoisomerases, blocking supercoiling catalyzed by DNA gyrase (in Gram-negative bacteria) and the development of the double helix mediated by topoisomerase IV (in Gram-positive bacteria). Blocking such mechanisms leads to the death of the bacterium. In addition, zoliflodacin stabilizes the enzyme–DNA complex for both gyrase and topoisomerase IV. In particular, the primary target of the antibiotic is the GyrB subunit of gyrase, unlike ciprofloxacin, which instead fits mainly into the pocket present in the GyrA subunit (bond with Ser83 and Asp87) [13].

*N. gonorrhoeae* mutants resistant to zoliflodacin are not observed, even when bacteria are exposed to combinations between zoliflodacin and antibiotics already in use, such as ceftriaxone, doxycycline, and gentamycin. Zoliflodacin does not show the phenomenon of cross-resistance with fluoroquinolones currently on the market [13]. It also has activities against *S. aureus* (MRSA), *Mycoplasma genitalium*, *Moraxella catarrhalis* (MIC90 of 0.25 μg/mL), vancomycin-resistant *H. influenzae* (MIC90 of 0.5 μg/mL), and *Enterococcus faecalis* (MIC90 of 1 μg/mL). Combining zoliflodacin with other antibiotics such as tetracyclines, ceftriaxone, or gentamycin increases its effectiveness against *N. gonorroheae*. Zoliflodacin has significantly lower MIC values with respect to other antibiotics on the market, with higher percentages of susceptible bacterial strains [15].

The in vitro tests to measure the inhibition of growth of myeloid and erythroid cell lines in mammals showed no genotoxicity or bone marrow toxicity for zoliflodacin at the highest concentrations tested. This suggests that this antibiotic presents reduced risks of hematologic toxicity compared with some fourth-generation fluoroquinolones (such as gemifloxacin), which have been associated with bone marrow suppression, anemia, and leukopenia. In addition, it has been shown that zoliflodacin is a powerful and very selective inhibitor of bacterial topoisomerases [13].

Two Phase I studies were conducted by administering individual doses for zoliflodacin to healthy volunteers [16]. The first study concerned the safety and tolerability of the molecule; the second focused, instead, on pharmacokinetic parameters (ADME). Phase II studies involved administering the antibiotic (single dose of 2 or 3 g) to men and women aged 18 to 55 with symptoms of urogenital gonorrhea. The data revealed the effectiveness of zoliflodacin against rectal infections, urogenital infections, and gonorrhea. The company *Entasis Therapeutics* announced the entry of the molecule into Phase III in 2019; the final results are expected in 2021 [10]. Another antibiotic inhibitor of topoisomerase II, gepotidacin, active against Gram-positive and Gram-negative cocci, is currently in Phase III for the treatment of urinary infections and gonorrhea [10].

### 4.2. C. difficile Infection: Focus on the New Compounds Ridinilazole and Bezlotoxumab

*C*. *difficile* infection (CDI) is the most common health-related nosocomial infection in the world, associated with high hospital costs. This is a looming threat to public health: It was estimated that in 2014, the costs of hospitalizing patients with recurrent CDI in the United States reached $6500 a day [17], which makes us aware of the enormous scale of the problem. Such infections are frequent, especially in nursing homes and stays for the elderly. After an initial episode of CDI, the risk of recurring infections developing increases exponentially; it can occur in 35%–65% of patients who have already contracted the pathogen [18]. Even after a course of therapy with metronidazole or vancomycin, 25% of patients suffer from a second infection. The patient is therefore in a sort of vicious cycle with continuous relapses: Those who have had an infection previously are much more predisposed to develop others later. Primary *C. difficile* infection is associated with a mortality range of 3 to 36%, values that rise by 33% in the case of recurrent infections [19].

The guidelines provide numerous treatments for primary infection, but options for the treatment of recurrent infections appear very limited. The objective in the clinical field is the management of the prevention of such relapses. More specifically, *C. difficile* is an anaerobic, Gram-positive, spore-forming, toxin-producing bacillus, causing infections ranging from medium-sized diarrhea to much more complicated and severe diseases such as enterocolitis and pseudomembranous colitis. It is present in healthy gut and vaginal microbiota. The spread of spores by symptomatic and asymptomatic patients determines the rapid spread of the pathogen. The spores, resistant to heat, radiation and alcohol-based disinfectants, are in fact persistent: The introduction of such spores by oro–fecal means is a kind of a foreman towards gut colonization by *C. difficile*. Spores travel from the stomach to the intestine where, under optimal conditions (high levels of colic acid in bile salts and lower levels of chenodesoxycholic acids), they turn into vegetative cells, which then colonize and proliferate in the colon, starting the infection itself.

A natural and innate defense against *C. difficile* is gut microflora. However, the imbalance in microbiota that usually occurs after a course of antibiotics predisposes to the occurrence of infection. The main condition, which allows the development of CDI, is in fact linked to the destruction of the normal microbial flora of the colon due not only to antibiotic therapy but also chemotherapy.

All classes of antibiotics are predisposing factors for the development of CDI, however antibiotics such as ampicillin, cephalosporins and clindamycin are the most risky for the onset of CDI. The best strategy for eradicating the bacterium would be to discontinue ongoing antibiotic therapy, although this is not always possible. Metronidazole (belonging to the nitro-imidazole class) has for years been the first-line treatment in the care of primary and non-severe CDI, but frequent resistance and increased failure rates, with an average of around 25% and peaks of 50%, have made it less effective [20]. Currently, vancomycin (a glycopeptide) is the preferred antibiotic in the treatment of both primary and recurrent infections. Despite this, the two drugs still showed negative effects on the gut microbiota. Another compound, fidaxomicin, represents a powerful option with minimal risk of damage to bacterial microflora [21]. However, the very high costs of treatment with fidaxomicin drastically limit its use in hospitals.

**Figure 2:**
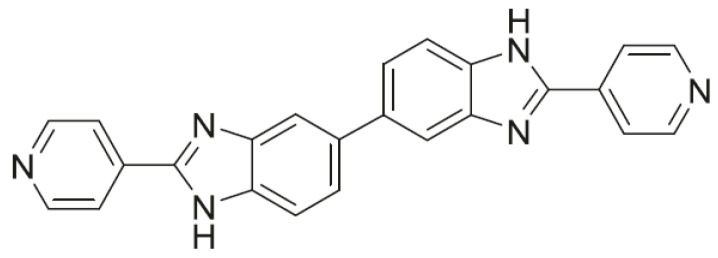
Formula of ridinilazole.

Other antibiotics used as the last possible option are tigecycline, teicoplanin, rifaximine, and bacitracin. There are now six new antibiotics under development versus *C. difficile*, the most promising being ridinilazole (Figure 2), in Phase III of clinical development. The common problem is always the treatment of recurrent infections, for which few compounds are really active. Clinical evidence also demonstrates the effectiveness of other therapies such as immunotherapy and fecal transplantation (fecal microbiota transplant), for which further studies are needed to confirm effectiveness [10].

Ridinilazole is a synthetic antibiotic of the class of bis-benzimidazoles, discovered by Summit Therapeutics for the treatment of *C. difficile*: It showed rapid bactericidal activity [22]. From the promising results of Phase II, it was evident that patients who were given ridinilazole had a greater clinical response in the eradication of *C. difficile* compared to patients who received vancomycin. From a chemical point of view (Figure 2), it is composed of a double benzimidazole (hence the name of the bis-benzimidazoles class), each one bonded to a pyridinic ring.

Ridinilazole has a unique mechanism of action: It probably inhibits the cell division of the bacterium, binding to the minor groove of DNA. Transcriptome analyses have, in fact, highlighted an altered expression of *C. difficile* genes involved precisely in cell division following exposure to the antibiotic [22]. After oral administration, it is poorly absorbed by the gastrointestinal lumen. Selective activity against *C. difficile,* demonstrated in vitro, together with limited systemic absorption and reduced action against gut microflora, make ridinilazole almost an ideal drug for the treatment of CDI.

MIC values were lower than metronidazole and vancomycin, comparable to those of fidaxomycin. No ridinilazole-resistant strains were highlighted. The results related to the activity on the microbiota were very positive, as the microflora remained almost unchanged after treatment with this drug, unlike vancomycin therapy (which led to a drastic decrease in *Bifidobacteria*) or the newest fidaxomycin therapy. In in vitro models of *C. difficile*-affected bowels, the antibiotic also showed only one activity against toxins A and B produced by the bacterium as well as decreased levels of interleukin (IL)-8. This is an important advantage; it means that it is potentially able to reduce the gut inflammation of the patient (present in the most severe forms of CDI). This is not the case in treatments with metronidazole and vancomycin, which have no action against toxins produced by gut cells. Phase II studies investigated the efficacy and safety of the antibiotic, always compared with vancomycin and metronidazole: Ridinilazole had side effects (especially in the gastrointestinal tract) comparable to those of vancomycin, but to a lesser extent. Phase III results (coming in 2021) will help outline the use profile and value of this drug.

In the 2019 analysis “Antibacterial agents in clinical development: An analysis of the antibacterial clinical development pipeline”, by the WHO [10], ten biological drugs are reported, including monoclonal and polyclonal antibodies used as a support for existing therapies; however, their potential uses in mono-therapy have yet to be investigated. The only monoclonal antibody, whose target is *C. difficile*, included in the report, is bezlotoxumab, approved by the FDA in 2016 and now marketed in the United States under the name Zinplava^®^ [17].

Recent studies have focused on new compounds preventing recurrent CDI, for which valid alternatives are still lacking, especially those targeting the virulence factors involved in the pathogenicity of the infection. The most severe forms of CDI are regulated by the expression of genes that control the main functions of toxin production (toxins A and B genes), toxin expression (toxin R), the release (toxin E), and toxin synthesis (toxin C) [23]. The production of toxins is the virulence factor that contributes most to the infection. Non-pathogenic strains of *C. difficile* produce spores but do not cause symptomatic infections. In symptomatic infections, vegetative cells release toxins, resulting in CDI.

Following the failure of actoxumab, a monoclonal antibody against toxin A, research has focused on compounds capable of blocking toxin B, which is responsible for the most severe pathological effects. Hence, bezlotoxumab (Zinplava^®^), a human monoclonal antibody directed against toxin B, showed promising results during Phase III. Bezlotoxumab is approved for the prevention of recurrent CDI in adults, administered in an intravenous formulation (10 mg/kg infusion as a single dose) in combination with an antibiotic treatment against *C. difficile*; it is, in fact, not effective in monotherapy. The results of Phase I and Phase II clinical trials showed significant benefits and reduced incidence of recurrent CDI (decreased by 40% in 12 weeks compared to placebo) [17]. No adverse events have emerged in healthy volunteers, not even drug-resistant bacterial strains. Based on Phase III data, in 2016, the FDA approved the use of bezlotoxumab in combination with antibiotics in the prevention of recurrent CDI.

## 5. Main Agents That Gained Market Authorization between 2017 and 2020

As of 2017, eight new antibiotics have been approved by the FDA, including one for the treatment of multidrug-resistant tuberculosis. The full list can be found in the “Agents that obtained market authorization” section of the “Antibacterial agents in clinical development: An analysis of the antibacterial clinical development pipeline” [10]. Derivatives of existing antibiotic classes, such as the tetracycline derivatives eravacycline and omadacycline as well as new β-lactams, prevail by far. Most of the approved compounds target carbapenem-resistant *Enterobacteriaceae* and other pathogens (of high and medium priority) included in the WHO’s list. Both omadacycline and eravacycline are derivatives of tetracyclines. Omadacycline is a semisynthetic drug and has activities against Gram-positives, including difficult to eradicate MRSA and some Gram-negatives. It is approved in the treatment of community-acquired pneumonia (CAP). Eravacycline, on the other hand, is totally synthetic and approved in the treatment of complicated intra-abdominal infections. The results of further studies must be awaited to better delineate the clinical profile of these antibiotics. Furthermore, a promising new combination of β-lactam antibiotic and β-lactamase inhibitor, presenting activities against *K. pneumonia* carbapenemase (KPC), has been approved. This is the combination meropenem and vaborbactam (a β-lactamase inhibitor). However, new treatment options for carbapenem-resistant *A. baumannii* (CRAB) and carbapenem-resistant *P. aeruginosa* (CRPA) are still lacking. The sole antibiotic approved in recent years against tuberculosis is pretomanid, a nitroimidazo-oxazine developed by the TB Alliance organization.

It is, together with bedaquiline and linezolid, a completely innovative treatment for adult patients with extremely resistant tuberculosis (XDR) and pulmonary multidrug resistant (MDR) tuberculosis. Most antibiotics that have obtained trade authorization are effective in treating complicated urinary infections and intra-abdominal infections. Companies involved in the research for new antibiotics have highlighted some difficulties; an example is the case of Achaogen, a biotechnology company that developed the aminoglycosydic plazomicin, approved in 2018, and went bankrupt a few months later despite receiving $2.4 million from Boston University’s CARB-X project for the development of the new drug [24].

For all eight antibiotics newly authorized by the FDA, obviously, post-marketing data are not yet available, and further studies are needed to define their therapeutic profile and adequacy of use in particular categories of patients.

### 5.1. Tetracycline Derivatives: Eravacycline

Eravacycline is a fully synthetic fluorocycline belonging to the tetracycline class, developed by Tetraphase Pharmaceuticals and approved by the EMA and FDA in 2018, for the treatment of complicated intra-abdominal infections (cIAI). It is marketed under the name Xerava^®^. cIAI normally extend into the area of the abdomen (peritoneal cavity, mesentery) and result in localized or diffuse peritonytes [25]. Especially if not treated, such infections are associated with significant mortality, and it is in fact necessary to start antibiotic therapy as soon as possible, which becomes fundamental, and in some cases, lifesaving. Usually, a large number of enteric microorganisms responsible for symptomatology are involved, such as *Enterobacteriaceae* (*K. pneumoniae*, *E. coli*), *Enterococcus* spp., and *Bacteroides* spp. The increase in MDR pathogens belonging to the aforementioned species is a very serious global health problem that threatens the treatment of such intra-abdominal infections.

**Figure 3:**
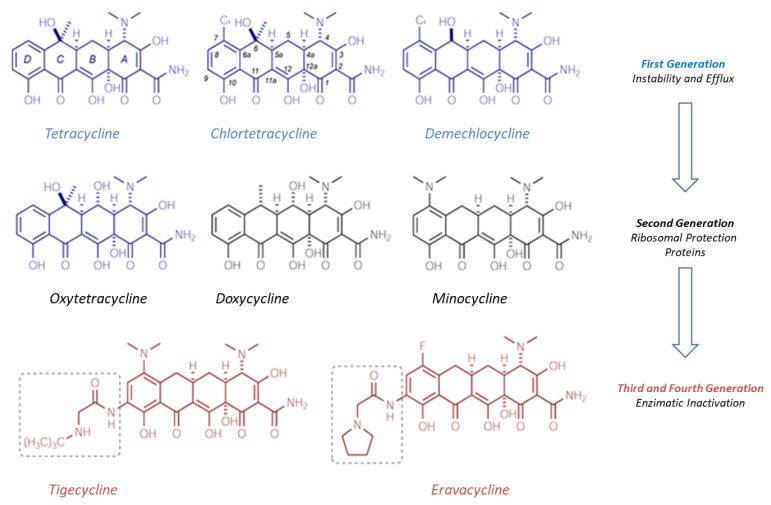
Different generations of tetracyclines and antibiotic resistance.

Eravacycline and relebactam with imipenem/cilastatin combination (Recarbrio^®^, approved in 2019) are included in the list of eight antibiotics authorized from 2017 to 2020 and constitute the most recent and innovative treatment options for patients with cIAI. Eravacycline was specifically developed to overcome the acquired resistance phenomenon experienced with traditional tetracyclines (Figure 3). The two primary mechanisms that confer the resistance of pathogens to tetracyclines are, in fact, the acquisition of genes encoding some efflux pumps and the presence of ribosomal protection proteins (RPPs) [26]. Various types of efflux pumps are present in the Gram-positive and Gram-negative bacteria; the most represented efflux pumps are encoded by tet(A) and tet(B) genes in Gram-negative and by tet(K) and tet(L) in Gram-positive bacteria.

First generation tetracyclines are more easily inactivated by efflux pumps in contrast to second generation tetracyclines (doxycycline and minocycline) or third generation tetracyclines (tigecycline), which are not sensitive to the actions of the pumps themselves. Efflux consists of actively reducing the concentration of the antibiotic within the bacterial cell thanks to the inducible synthesis of membrane proteins encoded by genes (tetA and tetB) placed on plasmids or transposons.

These proteins weaken the interactions between the tetracyclines and the binding site on the 30S ribosomal subunit. In fact, tetracyclines act by inhibiting protein synthesis, blocking the transfer of acyl-tRNA to that subunit. RPP also makes pathogens resistant to first and second generation tetracyclines, with less effect on the antibacterial activity of the latest generation tetracyclines. There are also other mechanisms of acquired resistance to tetracyclines such as mutations in the 16S RNA subunit; however, they are much less common than efflux pumps and ribosomal proteins.

Third generation tetracyclines (also called glycylcyclines), which include tigecycline and the new eravacycline, allow for overcoming the main resistances to tetracyclines: Efflux pumps do not recognize these molecules, as they have a substituent in position 9 of the tetracycle (Figure 3). This is the key difference from previous generations of tetracyclines. Moreover, they are also insensitive to the action of ribosomal protection proteins.

Eravacycline retains the pharmacophore characteristic of tetracyclines; however, it exhibits two unique changes in ring D at position C7 (addition of a fluorine atom) and at C9 (addition of a pyrrolidine acetamide group) [27]. The fluorine is not present in the tigecycline structure, which has a tertiary amino group in its place.

As a result of such substitutions in positions 7 and 9, eravacycline has activities against Gram-positive and Gram-negative bacterial strains that, in vitro, resulted in various mechanisms resistant to first- and second-generation tetracyclines. Like other tetracyclines, eravacycline performed its antibacterial activity by reversibly binding to the ribosomal subunit 30S, blocking the entry of molecules of the aminoacyl-tRNA complex. Compared to traditional tetracyclines, however, the link of eravacycline with ribosome is much stronger because it is able to recognize multiple attack sites, stabilizing the complex that forms. The compounds belonging to the first and second generation are bacteriostatic; however, eravacycline also has in vitro bactericidal activity against certain strains of *A. baumannii*, *E. coli*, and *K. pneumoniae*.

Eravacycline showed potent in vitro activity as determined by MIC90 against a broad spectrum of Gram-positive pathogens, including *E. faecalis* and *E. faecium*, both resistant to vancomycin, and *S. aureus* (MRSA), as well as against Gram-negative pathogens, including carbapenem-resistant *Enterobacteriaceae* [28]. Such pathogens are among those responsible for cIAI, so the drug has proven effective in clinical trials. Promising is the detected activity of eravacycline against isolated strains of *A. baumannii* MDR and resistant to carbapenems.

The MIC90 values of eravacycline measured for numerous Gram-positive and Gram-negative pathogens are clearly lower than those of antibiotics such as imipenem and vancomycin, remaining lower even in comparison with tigecycline. In addition, there is no cross-resistance mechanism between eravacycline and other classes of antibacterials such as fluoroquinolones, penicillins, cephalosporins, and carbapenems. The antibiotic is metabolized mainly by CYP3A4; therefore, the concomitant intake of eravacycline with strong inducers of this cytochrome (rifampicin, phenobarbital, carbamazepine, phenytoin, to name a few), accelerates the metabolism of the antibacterial drug, decreasing its plasma concentration.

Eravacycline in intravenous form has been approved since 2018 as Xerava^®^ in numerous countries, including the United States and some European states, for the treatment of cIAI, in adult patients. The recommended dosage is 1 mg/kg administered every 12 h for 4 to 14 days depending on the prescribed therapy [29]. In two double-blind clinical trials, the efficacy of eravacycline was not inferior to that of ertapenem and meropenem in terms of clinical response and acceptability. High doses of intravenous tetracyclines can cause microvescicular liver steatosis with lactic acidosis and severe liver dysfunction (LASH syndrome), but this complication has not been reported with the use of third-generation intravenous tetracyclines (eravacycline, tigecycline, omadacycline).

Given the wide spectrum of activity against clinically relevant common pathogens (including those expressing mechanisms of acquired resistance to tetracyclines) and the increased in vitro potency along with a better tolerability profile compared to tigecycline, eravacycline is an innovative option for the treatment of cIAI, especially against bacterial species resistant to traditional antibiotics.

### 5.2. Fourth-Generation Fluoroquinolones: Delafloxacin

Fluoroquinolones are effective antibiotics, used in therapy for over 50 years. However, the increase in resistance cases and some recorded adverse effects have severely limited their use. The last approved fluoroquinolonic, delafloxacin, is the only anionic (non-zwitterionic) antibiotic in this class. The particular molecular structure of the drug has given greater in vitro activity against many Gram-positive pathogens, including quinolone-resistant strains.

**Figure 4:**
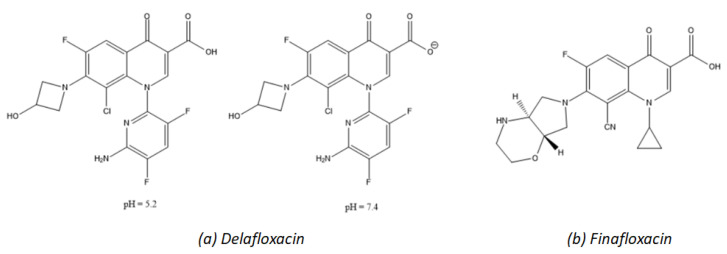
(**a**) Influence of pH on the chemical formula of delafloxacin. (**b**) Finafloxacin.

Delafloxacin (Figure 4) was developed by Melinta Therapeutics and then approved by the FDA in 2017 for the treatment of acute bacterial skin and skin structure infections (ABSSSI), marketed under the name Baxdela^®^. Such infections are associated with significant morbidity and mortality. Numerous Gram-positive and Gram-negative bacteria have been identified as etiological agents. However, the most dangerous pathogen for ABSSSI worldwide, is *S. aureus*, followed by other Gram-positive (*Enterococcus* spp., *Streptococcus pyogenes*) and Gram-negative (*P. aeruginosa* and *E. coli*) bacteria, more commonly found in surgical site infections [30]. A serious problem is the presence of pathogens resistant to traditional antibiotics, especially MRSA *Staphylococchi*: This contributes to the increase in mortality as well as the hospital costs related to such infections. Current guidelines in treatment offer a variety of antibiotics and therapeutic strategies, depending on the type—purulent (abscesses) or non-purulent (erysipelas, necrotizing infections)—and the severity of infection.

Usually, in the treatment of infections caused by methycillin-sensitive *S. aureus* (MSSA), it is recommended to use oxacillin or other β-lactamases-resistant penicillins or cephalozoline in the case of specific allergy to penicillins. If the etiological agent is MRSA, other more powerful antibiotics are administered such as vancomycin, linezolid, daptomycin, or ceftarolines. Sometimes, in cases of both MRSA and MSSA, more “dated” antibiotics are also used: clindamycin, minocycline, or the combination trimetoprim-sulfametoxazole. However, all these antibiotics are associated with limitations such as high levels of resistance (clindamycin and minocycline), high hospital costs and possible toxicity (linezolid), decreased sensitivity that involves using higher doses (vancomycin), and increased risk of developing *C. difficile* (clindamycin) infections. Therefore, new active antibiotics against resistant pathogens that cause ABSSSI have been studied, especially for infections caused by MRSA. The most recently approved antibiotics include dalbavancin, tedizolid, oritavancin, and delafloxacin. Another concern is *S*. *aureus’* ability to survive in the acidic environment of the skin.

The survival of bacteria depends on the expression of an enzyme that gives resistance to polyamines (anti-inflammatory compounds promoting tissue regeneration and wound healing). Polyamines are present in the skin acid environment and are toxic to *Staphylococcus*. Moreover, the bacteria are able to adopt specific behaviors that play an important role in the pathogenesis of infections such as their organization in biofilm [31].

The consequence is that the requirements of the new compounds are not only their activity against resistant strains but also their stability in the acid pH of the skin.

Delafloxacin has an increased activity in acidic mediums [32]. In addition, it shows promising efficacy on a wide spectrum of Gram-positive and Gram-negative bacteria involved in major acute skin infections. Delafloxacin differs from other fluoroquinolones in the absence of a basic group in position C7; as a consequence, this molecule is a weak acid, and at a neutral pH, it is an anion and not zwitterion, as are most of the antibiotics belonging to the same class. Moreover, in position C8, a chlorine atom is added, which acts as an electron-attractor group on the aromatic ring, improving polarity to the compound as well as increased activity and stability.

Finally, thanks to the voluminous heteroaromatic substitution in N1 (instead of the cyclopropyl present in ciprofloxacin and moxifloxacin), delafloxacin has a larger molecular structure compared to that of other fluoroquinolones. Due to the lack of a basic group in C7, the only ionizing group is the carboxyl in C3. At a neutral pH of 7.4, the predominant form (98.5%) is anionic delafloxacin (COO^−^), while at a slightly acidic pH of 5.2, the neutral form prevails (62.7%) (Figure 4a) [32].

These modifications have a direct impact on the activity of the antibiotic and may explain the increased potency at an acidic pH compared to other fluoroquinolones (second and third generation: ciprofloxacin and levofloxacin, but also fourth generation: moxifloxacin), for which activity decreases drastically in an acidic medium.

Delafloxacin also has lower MIC values than those of traditional fluoroquinolones against a wide spectrum of Gram-positive pathogens. Prior to delafloxacin, the most-recent fluoroquinolonic antibiotic was finafloxacin (Figure 4b), which was approved in 2014 for the treatment of acute otitis and has substantial differences compared to delafloxacin: It changes the group in C7 (more basic), there is no chlorine atom in C8, and it retains the cyclopropyl in N1 as in other fluoroquinolones.

Quinolones inhibit bacterial DNA and topoisomerase IV. The structural peculiarities of delafloxacin allow it to bind with equal affinity both to DNA gyrase and topoisomerase IV of Gram-positive (*S. aureus*) and Gram-negative (*E. coli*) bacteria. This reduces the likelihood of resistance, which requires the accumulation of multiple mutations at the level of both enzymes.

In the study promoted by CLSI (Clinical and Laboratory Standards Institute) and EUCAST (European Committee on Antimicrobial Susceptibility Testing) in 2016, it was found that delafloxacin had the lowest MIC values toward MSSA, MRSA, and *S. aureus* compared to other antibiotics such as levofloxacin, ceftaroline, ciprofloxacin, clindamycin, linezolid, and oxacillin [33]. Delafloxacin showed MIC values equal to the fifth part of those of ciprofloxacin against *Enterobacteriaceae* (*E. coli*) isolated from the urine of patients suffering from urinary infections. Another in vitro study showed the efficacy of delafloxacin in combination with caspofungin against many Gram-positive infections. Caspofungin is an antifungal drug inhibiting the synthesis of the polysaccharide components of the bacterial biofilm of *S. aureus* [34]. As mentioned before, delafloxacin is active at an acidic pH. This has been demonstrated by comparing this antibiotic and other fluoroquinolones at several pH values. Moreover, in vitro studies have confirmed the effectiveness of delafloxacin in fluoroquinolonic-resistant bacterial strains: It is bactericidal against *E. coli* in 6 h and *S. aureus* in 10 h. It has also been found to be more active than other antibiotics against Gram-negative pathogens such as *H. influenzae*, *N. gonorrhoeae*, *Legionella* spp., *P. aeruginosa*, and *H. pylori* [32].

Fluoroquinolones have collected a long history of adverse events, including tendinitis, swelling and tendon injuries, memory problems, muscle pain or weakness, peripheral neuropathy, and exacerbations of myasthenia gravis. As a result, in the United States, many fluoroquinolones on the market, including delafloxacin, carry a boxed warning on the outer packaging and in the package leaflet about these effects [35]. The EMA has also added restrictions on the use of such antibiotics, which must be administered only to particular infections (to “severe infections for which no other antibiotics can be used”) [36].

The FDA reported that peripheral neuropathies and effects on the nervous system were observed during the clinical development of delafloxacin as well as diarrhea associated with *C. difficile*; however, these effects were not recorded more frequently in the cohort receiving the antibiotic than in the comparison cohort [37]. Numerous studies have been carried out to examine the specific side effects related to fluoroquinolone class membership. In clinical models, delafloxacin, compared to moxifloxacin, has not been related to heart disorders (such as QT stretching) even at doses higher than therapeutic ones [38]. Moreover, another study was conducted on photosensitivity, commonly associated with the presence of the halogen substituent in position C8 (as it is for lomefloxacin, which has a fluorine in C8), in which delafloxacin was found not to be phototoxic, despite the presence of a chlorine atom in C8 [39]. However, the safety profile of delafloxacin will be further demonstrated through large-scale use.

Based on pharmacological data, delafloxacin (Baxdela^®^) shows favorable properties: it maintains 60% bioavailability after oral administration, it is a mild inhibitor of cytochrome P450 3A, it interacts with few other drugs, and there is no cross-resistance with fluoroquinolones currently on the market. It is available in both tablets and an intravenous formulation for the treatment of acute skin infections, and it could also be effective in respiratory infections. Of course, this must be confirmed by further studies in the coming years.

### 5.3. New Β-Lactams in the Treatment of Multidrug-Resistant Enterobacteriaceae Infections: The Meropenem–Vaborbactam Combination

Recently, there has been a continuous increase in the number of infections due to Gram-negative pathogens, especially carbapenem-resistant *Enterobacteriaceae*, included by the WHO in the list of critical priority pathogens that pose a looming danger to world health. The family *Enterobacteriaceae* consists precisely of Gram-negative bacteria whose natural habitat is the animal intestine and they are responsible for a long list of gut and urinary infections. The main mechanism of resistance of these pathogens to many traditional antibiotics is attributable to the production of carbapenemases, or enzymes that include a variety of beta-lactamases capable of hydrolyzing both carbapenems and penicillins, some cephalosporins, and aztreonam. The activity of these enzymes, with rare exceptions, is not blocked by classic β-lactamase inhibitors such as clavulanic acid, tazobactam, and sulbactam. KPC is the most-frequently produced enzyme by pathogens belonging to the *Enterobacteriaceae* species. Such CRE-producing pathogens are often isolated from patients’ urine, which is not surprising, as *Enterobacteria* are responsible for most of complicated urinary tract infections (cUTI), especially those associated with high mortality. In fact, invasive infections caused by CRE result in mortality of between 26% and 44% [40]. The production of carbapenemases is not the only mechanism that *Enterobacteria* possess to develop resistance: There are also efflux pumps, enzymatic degradations, mutations at the porine level, and alterations of the target site. As a result, treatment options for CRE infections are unfortunately limited. In some cases, no first-line antibiotics are active against such pathogens, and the only treatments available are polymyxins and aminoglycosides or rather old antibiotics in addition to the rediscovery of colistine, the toxic effects of which are by no means negligible. It is clear that new compounds are needed to treat Gram-negative bacteria infections, mainly CRE.

Β-lactams are a class of antibiotics with absolutely established use. They attack peptidoglycan biosynthesis, interrupting the formation of the bacterial cell wall through covalent binding to PBPs. The group includes penicillin, cephalosporins, carbapenems, and monobactams. The emergence of β-lactamase-producing bacteria has made many of these antibiotics ineffective; moreover, the spread of extended spectrum β-lactamases (ESBLs) also gives resistance to third-generation, broad-spectrum cephalosporins such as ceftriaxone and ceftazidime. Class B β-lactamases contain a zinc ion at the active site of the enzyme. The other classes of β-lactamase (type A, C, and D) are serine β-lactamases. The main strategy to stem the hydrolysis of antibiotics belonging to this class is to combine a β-lactam and a β-lactamase inhibitor (BLI) such as clavulanic acid, tazobactam, or sulbactam.

The latter are able to inhibit the aforementioned ESBLs; however, they have no activity towards carbapenemases. Recently, some combinations of β-lactamase inhibitors with carbapenems or cephalosporins have been approved, including ceftolozane with tazobactam and ceftazidime with avibactam. Taniborbactam/cefepime (in clinical development) and cefiderocol (already approved) cover all classes of β-lactamases, including class D, produced by *A. baumannii* [10].

**Figure 5:**
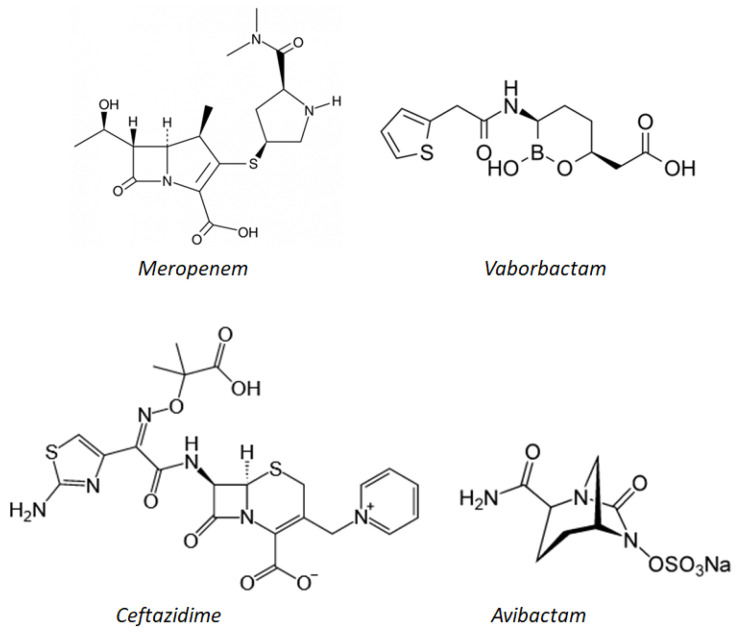
Meropenem/vaborbactam and ceftazidime/avibactam.

In 2015, the combination of ceftazidime (a broad-spectrum, third-generation cephalosporin) with avibactam (Zavicefta^®^ and Avycaz^®^) (Figure 5), was approved for the treatment of cUTI and cIAI. This combination is active in vitro and inhibits class A (e.g., KPC) and Class D (e.g., OXA-48) carbapenamases.

Retrospective studies have shown a decrease in mortality from CRE infections and an increased survival rate of 92% with ceftazidime/avibactam, compared to 55% mortality observed using the combination of colistine, aminoglycosides, and carbapenems [41].

Although these data are promising and encouraging, cases of resistance in *Enterobacteria* treated with ceftazidime and avibactam are already reported. This shows the huge need for new active compounds against CRE along with conscious and appropriate use of existing antibiotics.

The combination of the carbapenemic antibiotic meropenem and vaborbactam (Figure 5), a new β-lactamase inhibitor based on the boron acid formula, has powerful in vitro activity against *Enterobacteria* producing KPC [42]. In 2018, this association received marketing authorization for the treatment of cUTI, including acute pyelonephritis, cIAI, and hospital-acquired pneumonia (HAP), including assisted ventilation pneumonia (VAP). It was developed by Rempex Pharmaceuticals and marketed as Vabomere^®^.

From a chemical point of view, meropenem is a 1-β-methyl carbapenem. It is produced by total chemical synthesis. Unlike imipenem, it has a carbon methyl group (β) at position 1 as well as a different carbon substitution at position 2. The side chain linked to C2 is, in fact, much more cluttered than that of the imipenem. This justifies the greater stability of meropenem compared to hydrolysis by the enzyme human renal dehydropeptidase-1 (DHP-1), which is why it does not require co-administration with cilastatin (which was indispensable in the case of imipenem). Moreover, it is stable even in the presence of β-lactamases, including penicillinase and cephalosporinase, thanks to the presence of 6-trans-hydroxyethyl. Meropenem is marketed under the name Merrem^®^ for parenteral use. It has been authorized in the European Union since the 1990s [42].

Vaborbactam is a new inhibitor of β-lactamases whose cyclic pharmacophore is based on the structure of boronic acid. It strengthens the activity of meropenem alone. The boronic ester allows the compound to assume a particular conformation that can selectively inhibit β-lactamases as compared to mammalian serine-proteases. In particular, the portion derived from boron mimics the tetrahedral intermediate that is formed as a result of the interaction between the hydrolytic enzymes such as metallo-β-lactamases (class B) or serine β-lactamases (class A, C, and D) and the β-lactam antibiotic. In this way, the enzyme binds to vaborbactam instead of inactivating the antibiotic. In vitro experiments were conducted to explore the SAR of vaborbactam with the aim of finding the best substitutes to enhance the activity of meropenem: In particular, the addition of the thienyl-acetyl group in position 2 of the ring proved to be very promising.

Vaborbactam inhibits many class A and C β-lactamases and carbapenemases, and it is especially essential that it is effective against KPC. Vaborbactam manages to enter the outer membrane of the bacterium *K. pneumonia* by exploiting the porines OmpK35 and OmpK36 [43].

Meropenem is a broad-spectrum, bactericidal carbapenem with activity to many MDR pathogens, and it remains stable even in the presence of extended spectrum β-lactamases (ESBL). Vaborbactam alone, on the other hand, has no antibacterial activity. For strains of *Escherichia coli* that produce carbapenemases, the values of MIC for the combination meropenem with vaborbactam and for meropenem alone were both ≤0.03 mg/L [44]. The addition of vaborbactam did not improve the effectiveness of meropenem against *Acinetobacter* spp. or *P. aeruginosa* because the resistance of such bacterial species to carbapenems was multifactorial: It was not only caused by the production of β-lactamases but also depended on other mechanisms (one of them was the expression of efflux pumps). The combination showed, however, powerful in vitro activity against numerous strains of *Enterobacteria*, including carbapenem-resistant *K. pneumonia*. In fact, in the presence of CRE, vaborbactam greatly enhanced the effectiveness of meropenem alone.

On 9 July 2020, the R&D division of Menarini Ricerche Group announced the publication of an abstract that reported the latest evidence deriving from the clinical studies on meropenem/vaborbactam (marketed as Vaborem^®^ in the European Union and as Vabomere^®^ in the USA) [45]. Based on the TANGO I (Targeting Antibiotic Non-susceptible Gram-negative Organisms) clinical study, which compared meropenem/vaborbactam with the piperacillin-tazobactam association, Vabomere^®^ was initially approved by the FDA for cUTI, including pyelonephritis, in adult patients.

In this randomized Phase 3 study, Vabomere^®^ was administered in monotherapy to patients with confirmed or suspected CRE infections and was compared with the best available treatment, which consisted mainly of monotherapy or combinations of multiple antibiotics (polymyxin B, colistine, carbapenems, aminoglycosides, thygecycline, or ceftazidime/avibactam). During the study, the association of meropenem and vaborbactam showed a considerable reduction in mortality and an improvement in clinical safety (decreased adverse events, such as nephrotoxicity) and tolerability and was shown to be an effective therapeutic option for the treatment of HABP/VABP (bacterial pneumonia associated with the ventilator) and bacteriemia from CRE. Clinical studies have shown the good tolerability of the combination of meropenem and vaborbactam; the most common side effects recorded in TANGO I were headaches, diarrhea, and nausea.

Meropenem/vaborbactam could represent a turning point in the fight against Gram-negative infections that are difficult to treat, as it addresses the important medical issue of carbapenem-resistant *Enterobacteria*. It should be considered a first-line treatment for the treatment of infections from KPC-producing pathogens, with use restricted to these particular infections. Further results and future work will make it possible to define the role of this combination of antibiotics, which is certainly an additional weapon to combat the growth of resistance to carbapenems in *Enterobacteria* [42].

**Figure 6:**
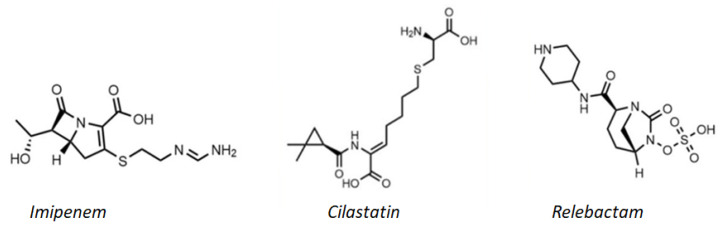
Imipenem/cilastatin/relebactam.

Relebactam is an active β-lactamase inhibitor against class A (including KPC) and class C β-lactamases. The structure is similar to that of avibactam. In vitro studies have shown that the addition of relebactam to the combination of imipenem/cilastatin (Figure 6) restores the activity of the same association against strains of *Enterobacteriaceae* that produce KPC, normally not sensitive to imipenem [42].

Phase II studies have shown the effectiveness and tolerability of the association of imipenem and relebactam in the treatment of cIAI, cUTI, and acute pyelonephritis. Phase III was completed in 2018 [42]. Developed by Merck & Co., the drug containing imipenem monohydrate, sodium cilastatin, and relebactam monohydrate is marketed in the European Union under the brand name Recarbrio^®^; this medicinal product requires additional clinical monitoring because of the absolutely promising in vitro results’ lack of extended clinical data. This combination could represent a valid alternative in the treatment of complicated, carbapenem-resistant *Enterobacteriaceae* infections, especially KPC producers, together with the aforementioned meropenem/vaborbactam association.

### 5.4. New Aminoglycosides in the Treatment of Infection Caused by Multidrug-Resistant Enterobacteriaceae: *Plazomicin*

Aminoglycosides are historical antibiotics, used in therapy for many years. They are irreversibly bound to a ribosomal site consisting of three proteins of subunit 50S (mechanism of action of streptomycin) and possibly other proteins of subunit 30S (all other aminoglycosides). As a result, they block the ribosome on the starting codon (AUG), which results in the detachment of the ribosomal complex and an incomplete synthesis of the protein. They are bactericidal antibiotics on Gram-negative aerobes and some Gram-positive and *Mycobacteria* spp. Parenteral use is limited to serious infections with Gram-negative bacteria and as antitubercular agents; in fact, many aminoglycosides have nephrotoxicity and ototoxicity when administered through this route. The onset of antibiotic resistant phenomena of this class is occurring more and more often. The most common resistance mechanism consists of the production of enzymes (acetyltransferase, phosphorylase, adenosyltransferase) that inactivate the antibiotic through conjugation reactions at the expense of amine and oxidyl functions, making it less akin to binding sites in the bacterial ribosome. Susceptibility to these enzymes is different in various aminoglycosides: It is minimal in amikacin and netilmycin (both of semisyntetic origin), thanks to the presence of substitutes that sterically interfere with the binding to the inactivation enzyme. There are also ribosomal modifications that produce high resistance: These are methylations of specific bases (guanine) of rRNA in subunit 16S. Enzymatic resistance to aminoglycosides is very common in the *Enterobacteriaceae* species.

**Figure 7:**
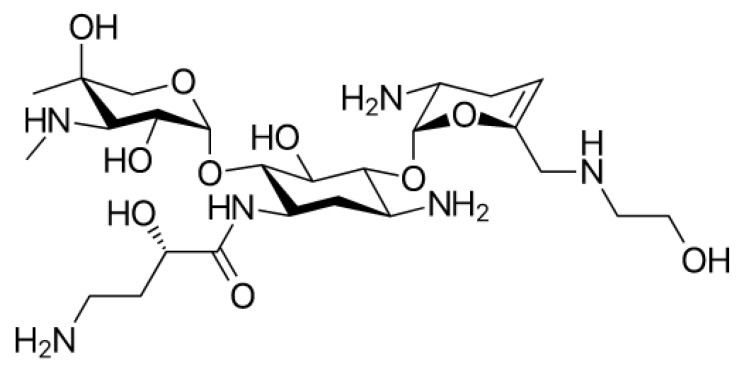
Formula of plazomicin.

Plazomicin (Figure 7) is a new aminoglycoside that derives from the modification of sisomicin (a specific antibiotic against Gram-negative infections for which gentamicin, the first-choice molecule, did not give the desired effects) [46]. Plazomicin, specifically in *Enterobacteria* spp., blocks most of the Aminoglycoside Modifying Enzymes (AME) inactivating aminoglycosidic antibiotics. This is due to the innovative chemical structure of plazomicin compared to other aminoglycosides: It differs considerably from the structures of gentamycin and tobramycin but gets closer to that of amikacin.

**Figure 8:**
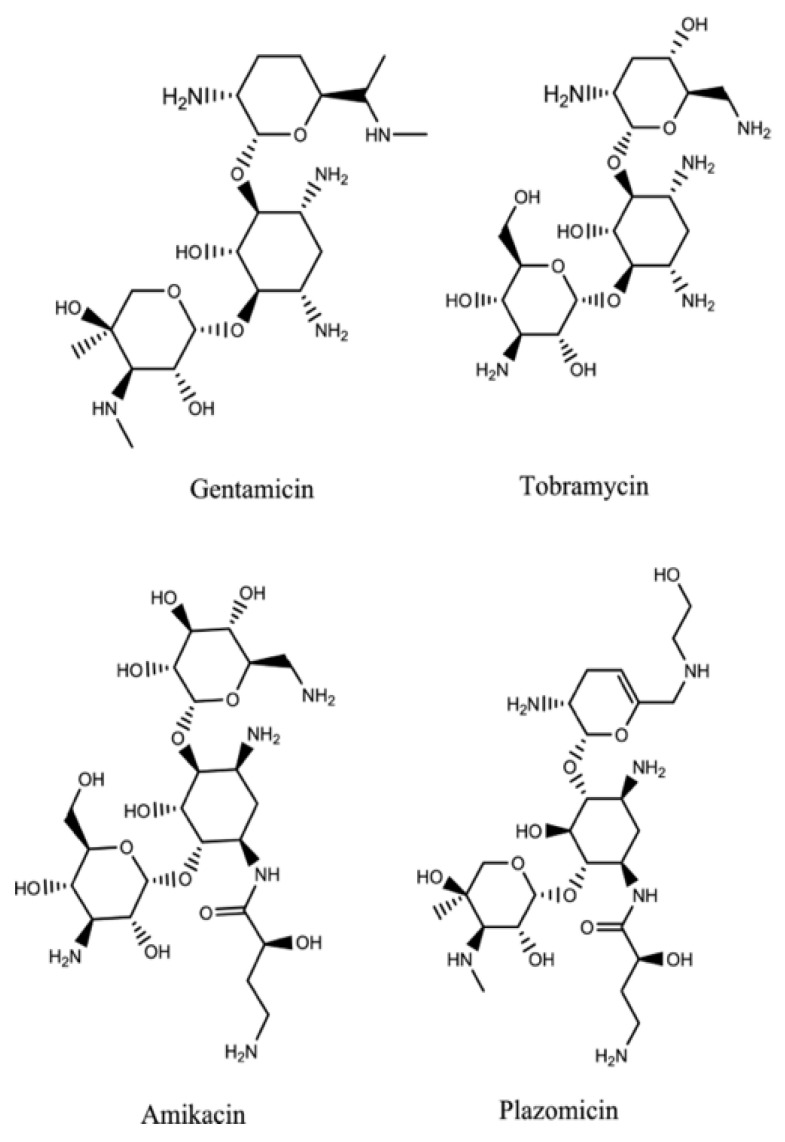
Group of 2-deoxystreptamines.

Plazomicin belongs to the group of 2-deoxystreptamines (Figure 8) in addition to amikacin, gentamycin, and tobramycin. As in amikacin and plazomicin, the aminocyclitol is substituted in positions 1’, 4’, and 6’. In particular, to block inactivating enzymes, the substituent (hydroxyethyl group) in position 6’, is bulkier compared with the other antibiotics of this class. Moreover, between positions 4′ and 5′, there is a double bond that is not present in the structures of other aminoglycosides except for netilmycin, an unsaturated derivative of gentamycin that is endowed with less ototoxicity.

In position 1, there is hydroxy aminobutyric acid already inserted in the structure of amikacin; it specifically prevents adenylation and phosphorylation, resulting in an increase in antibiotic potency and spectrum enlargement.

In vitro studies report that resistance to plazomicin may occur by methylation of ribosomal subunit 16S. Plazomicin is a broad-spectrum antibiotic, with activities against many Gram-positive and Gram-negative bacteria, including CRE and KPC *Enterobacteria*, which are pathogens that produce ESBL, and strains of *E. coli* not sensitive to aminoglycoside gentamycin [47]. It is more potent than antibiotics belonging to the same class against KPC *Enterobacteria*. In fact, among strains of KPC-producing *K. pneumoniae*, the measured values of MIC50 of plazomycin were 0.5 mg/L, while gentamycin had a MIC50 of 8 mg/L and amikacin and tobramycin more than 32 mg/L [48].

Plazomicin demonstrated promising efficacy and safety in Phase II results in the treatment of urinary infections, which allowed two Phase III studies to begin. The first is the EPIC clinical trial (Evaluating Plazomicin in cUTI) where plazomicin was administered in an intravenous formulation to adult patients hospitalized with cUTI or acute pyelonephritis, both caused by *Enterobacteriaceae*. The second is the CARE (Combating Antibiotic-Resistant *Enterobacteriaceae*) a randomized study evaluating the efficacy and safety of plazomicin-based combination therapy compared with colistin-based combination therapy for the treatment of patients with invasive, CRE-involved infections such as ventilator-associated pneumonia (VAP), hospital-acquired pneumonia (HAP), and cUTI. In both studies, plazomicin proved to be well-tolerated. There was, however, reversible ototoxicity in a patient involved in the EPIC study.

Developed by Achaogen, plazomicin was approved by the FDA in the United States in 2018 with the name of Zemdri^®^ as alternative for the treatment of cUTI and pyelonephritis caused by *Enterobacteriaceae* spp. (including *E. coli*, *K. pneumoniae*, *Proteus mirabilis*, and *Enterobacter cloacae*). In Europe, plazomicin has not yet received marketing authorization.

### 5.5. Siderophore Cephalosporins: Cefiderocol

Cephalosporins belong to the class of β-lactam antibiotics, and they were discovered in 1945 by the Italian, Giuseppe Brotzu, who was the rector of the University of Cagliari in Sardinia, Italy. The mechanism of action is identical to that of penicillins: They act by blocking the synthesis of the bacterial wall. There are five generations of cephalosporins, each characterized by a precise antimicrobial spectrum that becomes wider and wider reaching the fifth generation, also active on MRSA. In fact, the compounds belonging to the latter generation (ceftobiprole, ceftarolin, ceftolozane) have been developed to specifically combat MDR bacterial strains. Ceftobiprole, used to treat community-acquired pneumonia, is effective against methycillin-resistant *Staphylococchi*. Ceftolozane, combined with the β-lactamase inhibitor tazobactam (Zerbaxa^®^), is highly dedicated to carbapenem-resistant *Enterobacteriaceae* and *P. aeruginosa*.

Cefiderocol is part of the siderophore cephalosporins, a new class of drugs, of which this antibiotic was the first to be approved, by the FDA in 2019 and by EMA in April 2020, for cUTI caused by Gram-negative, community-acquired bacterial pneumonia (HABP) and ventilator-associated bacterial pneumonia (VABP) [49].

Siderophores are molecules with the marked properties of chelate ions, especially iron; they are produced and released by numerous bacterial species to facilitate the transport of ions into the cell, as required for supporting biological functions and bacterial growth. Siderophores all share the same structure: a functional unit that binds iron (transferrin or lactoferrin) and a peptide that interacts with a receptor present on the surface of the bacterial membrane.

**Figure 9:**
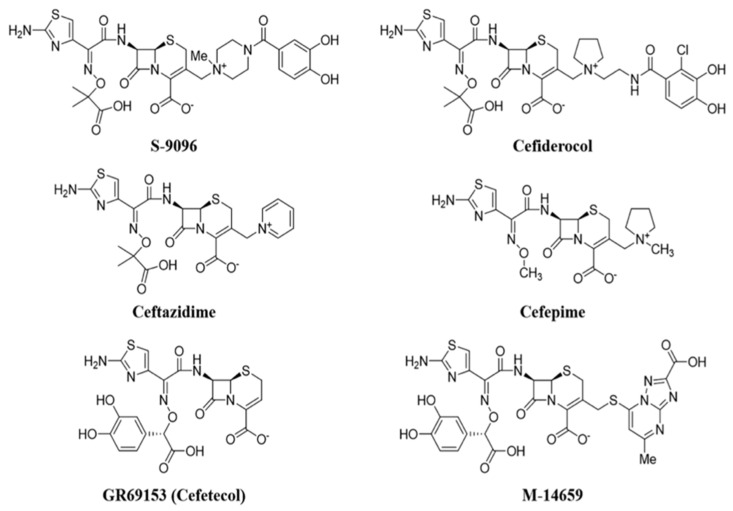
Structures of different cephalosporins conjugated with siderophores.

Research on siderophores has suggested that their involvement in the active transport of the bacterial cell makes pathogens more sensitive to antibiotics associated with siderophore groups; in fact, the measured MIC values are lower than traditional antibiotics. In the 1980s, researchers from some companies began to develop synthetic β-lactam antibiotics functionalized with siderophores, which showed powerful in vitro antibacterial activity against numerous Gram-negative bacteria, including *P. aeruginosa*. The siderophore group of these molecules seizes iron from the external environment. The iron–siderophore–antibiotic complex binds to the iron transporter outside the bacterial membrane, and it is actively transported inside the bacterial cell, bypassing the pathogen’s defense systems. This mechanism is called the “Trojan horse strategy” and allows for exploiting the iron transporter, improving the penetration of the antibiotic. In addition, the development of intrinsic and acquired resistance mechanisms is avoided. The first compounds involving the conjugation of cephalosporin–siderophore portions (Figure 9) such as cefetecol (GR69153) and M-14659 (specific anti-Pseudomonas cephalosporin) could not pass the early clinical stages, despite their powerful in vitro activity.

In the 1990s, the Japanese company Shionogi & Co developed the cephalosporin S-9096, which showed powerful activity against *P. aeruginosa*. This compound presents a new catechol moiety also found in natural siderophores produced by *E. coli* and *P. aeruginosa* (i.e., enterobactine and pyoverdine), however, S-9096 didn’t pass the clinical stages due to low stability and potential cardiotoxicity. Shionogi’s researchers initiated new research on siderophoric cephalosporins in the early 2000s, when antibiotic resistance had increased exponentially from 20 years earlier and few therapeutic alternatives were available. [49]. The challenge was to translate the great in vitro activity shown by the first siderophore cephalosporin into the development of products with in vivo activity and good pharmacokinetic and pharmacodynamic properties.

The SAR of natural and cephalosporin-conjugated siderophores leaded to the development of cefiderocol (S-649266) bearing a catechol moiety. In vitro studies have shown that this compound is up to 100 more stable to the action of different types of carbapenemases than ceftazidime.

**Figure 10:**
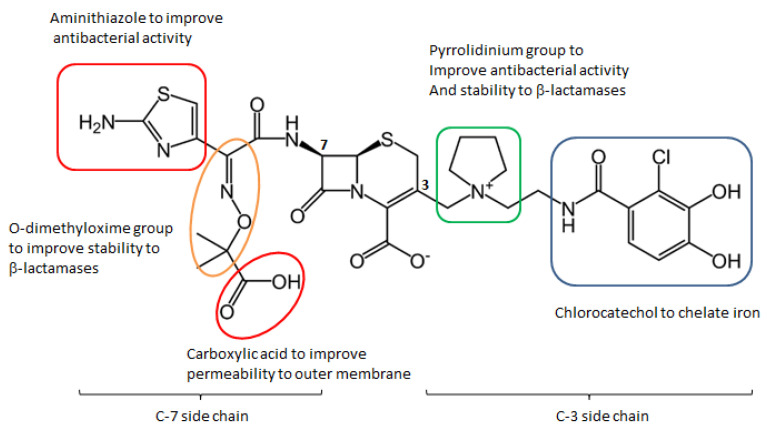
Cefiderocol: structure–activity relationships (SAR).

The structure of cefiderocol (Figure 10) is similar to that of cefepime, a fourth-generation cephalosporin: Both have a pyrrolydinic group bound to the chain in C3, which results in quaternary ammonium. They are zwitterions; this allows them to penetrate better into both the Gram-positive and Gram-negative. An additional (carboxypropyl)oximine chain and an aminothiazole ring (common to many broad-spectrum cephalosporins) increase their antibacterial activity to Gram-negatives. Carboxylic acid in the C7 side-chain improves the permeability of cefiderocol in the outer membrane. Oxime and dimetyl groups, on the other hand, increase stability toward hydrolysis by β-lactamases.

The main distinction between cefiderocol and the other cephalosporins examined (ceftazidime and cefepime) of previous generations lies in the substitute in position 3: this gives siderophore properties. Cefiderocol contains a portion consisting of a chlorocatechol (chloro-chloro-3,4-dihydroxibenzoic acid) covalently bound, via a particular linker, to the nitrogen of the pyrrolidine ring, to form a quaternary ammonium cation. The additional catechol portion allows achieving high plasma concentrations of cefiderocol compared to those of ceftazidyme and cefepime, thanks to the ability of the two hydroxyl groups to chelate the iron ion (Fe3^+^) and, consequently, exploit the transporter of the same ion [50]. All these structural changes compared with the oldest cephalosporines give cefiderocol a strong stability against β-lactamases, including carbapenemases, while maintaining a high affinity toward the molecular target, the PBPs. It is transported inside the bacterial cell through iron transport systems located on the outer membrane of gram-negatives. Once the complex has passed the outer membrane, cefiderocol dissociates and, like other β-lactam antibiotics, inhibits PBPs, resulting in the death of the bacterium that can no longer synthesize the cell wall. The active transport of the cefiderocol–iron complex not only contributes to making this antibiotic available within the periplasmic space where PBPs are located but also overcomes the problems related to the low permeability of the drug due to the bacterial outflow pumps that tend to expel it. In fact, cefiderocol also maintains effectiveness in cases of up-regulation of efflux pumps, which is one of the mechanisms of resistance developed by pathogens against carbapenemases.

A major point in favor of cefiderocol is structural stability against a wide range of serine and metallo-β-lactamases such as KPC, oxacillin carbapenemase (OXA), and New Delhi metallo-β-lactamase (NDM) [51]. The results of a study on this topic [49] showed that bacteria could potentially more easily develop resistance to ceftazidime and other third and fourth generation cephalosporins than cefiderocol, and there was no cross-resistance between it and the other cephalosporins.

Although rather limited information is still available, recent studies suggest that resistance to cefiderocol may occur due to genetic mutations at the iron carrier level; the molecular mechanisms underlying these considerations remain to be clarified [52].

Cefiderocol has an absolutely unique antibacterial spectrum against a wide variety of clinically relevant Gram-negative strains, including not only pathogens belonging to *Enterobacteriaceae* (*Klebsiella* spp., *Shigella flexneri*, *Salmonella* spp., *Vibrio* spp., and *Yersinia* spp.), but also against bacterial species such as *Acinetobacter* spp., *Pseudomonas* spp., and *Burkholderia* spp. [53]. It also has activities against pathogens such as *Haemophilus* spp. that cause respiratory tract infections. Moreover, this antibiotic shows powerful in vitro activity, with low MIC values on several multidrug-resistant Gram-negative strains and β-lactamase producers strains (including ESBL, serine and metallo-carbapenemase).

Cefiderocol is very promising, also, for the treatment of carbapenem-resistant strains, which are considered critical priorities by the WHO. This is demonstrated by the results reported in the Global Surveillance Study [54], which collected data from three consecutive SIDERO-WT annual studies from patients in Europe and North America.

Almost all (96.2%) isolated strains of *Enterobacteriaceae*, *A. baumannii*, *P. aeruginosa*, and *Stenotrophomonas maltophilia* are sensitive to less than 4 mg/L of cefiderocol. Gaussian distribution links the percentage of strains to the MIC value of cefiderocol.

Unlike previous siderophore cephalosporins, the very good in vitro activity of cefiderocol is supported by in vivo clinical efficacy. Consequently, cefiderocol has been approved as an injectable drug and in addition, is the first cephalosporin capable of treating *A. baumannii* infections. In 2019, the FDA announced its approval under the trade name Fetroja^®^ for the treatment of adult patients with cUTI, including kidney infections caused by sensitive, Gram-negative microorganisms, which have limited treatment options or no alternative. In 2020 the indication was added for the treatment of HABP and VABP caused by the following bacteria: *A. baumannii*, *E. coli*, *Enterobacter cloacae*, *K. pneumoniae*, *P. aeruginosa*, and *Serratia marcescens*. Following that, cefiderocol was also approved by the EMA in April 2020.

The safety and efficacy results of the pilot study on patients with cUTI showed that 72.6% had a resolution of symptoms and eradication of bacteria seven days after treatment as compared to 54.6% in patients who received an alternative antibiotic. Fetroja^®^ received from the FDA the designation of a Qualified Infectious Disease Product (QIDP), which is given to antibacterial and antifungal products intended to treat serious or life-threatening infections. The real novelty compared to the other newly approved antibiotics (including the combination already seen of meropenem/vaborbactam) is its ability to overcome all three mechanisms of development of resistance to β-lactams, namely the production of bacterial β-lactamases, the up-regulation of efflux pumps, and the modification of porines.

### 5.6. Treatment of Multidrug-Resistant Tuberculosis: Pretomanid

Tuberculosis is an infectious disease caused by *Mycobacterium tuberculosis* (Koch bacillus); it is categorized as pulmonary tuberculosis, generalized tuberculosis (tubercular sepsis), and extra-pulmonary tuberculosis. *Mycobacteria* are acid-resistant bacilli with an elaborate wall that are characterized by the presence of several unusual lipids. It is believed that, to date, approximately a third of the world’s population suffers from latent tuberculosis, and in addition, about 2 million people die each year as a result of infection [55]. Tuberculosis is also the leading cause of death in individuals with HIV. Of the 10 million TB cases recorded in 2019, at least 500,000 were resistant to rifampicin or the rifampicin–isoniazide combination, using two of the most widely used frontline drugs. The biggest problem at present lies in the presence of multidrug-resistant forms of tuberculosis (MDR-TBC), with a mortality rate approaching 50%. Numerous awareness campaigns have been conducted, including the “Stop TB” strategy of the World Health Organization, which aims, by 2030, to eradicate the tuberculosis epidemic. Achieving this will be extremely challenging, but also stimulating. To eradicate mycobacterium, it is necessary to use therapies combined with at least two drugs to which the bacillus is sensitive in order to reduce the selection of mutant strains and, at the same time, generate a synergistic effect. Combined therapy should continue for a long time, for a minimum of 6 months, with an inevitable incidence of side effects and frequent interactions with other drugs. If the first-line drugs (isoniazid, rifampicin, ethambutol, pyrazinamide) have not been effective, especially due to the onset of resistance phenomena, second-line drugs (para-aminosalicylic acid, ethionamide, thyoacetazone, amikacin, and many others) are used. Unfortunately, there are many forms of TBC resistant to conventional treatment that constitute a real threat to world public health. Resistant tuberculosis is classified as MDR-TBC when there is no response to rifampicin and isoniazid (frontline drugs); extensively resistant (XDR-TBC) in the event that the administration of three or more second-line drugs is not effective (generally not resistant to fluoroquinolones and at least to another second-line injectable drug); and totally drug-resistant (TDR-TBC), i.e., not treatable with any of the drugs that currently exist. The treatment of MDR-TBC consists of taking multiple drug therapy for a period of at least 21 months. Aminoglycoside antibiotics such as capreomycin and kanamycin can be used as well as fluoroquinolones such as ofloxacin and moxifloxacin and in some cases, cycloserine as well. The antibiotic linezolid (oxazolidinone) is often prescribed in severe cases of multidrug-resistant tuberculosis, but there are numerous side effects related to the drug.

It is clear that, especially for the most difficult forms of tuberculosis to treat, new drugs are needed that also manage to reduce the overall duration of treatment and are also compatible with antiretroviral drugs administered to HIV-positive patients who contract a *M. tuberculosis* infection. Currently, 12 new active ingredients against *M. tuberculosis* are in clinical development, 7 of which meet the criterion of innovation that provides for the absence of cross-resistance, while 6 antibiotics are able to meet all four criteria. Eight promising compounds are in Phase II and III [10].

The molecular targets that these drugs inhibit are multiple and diverse: the enzyme DprE1 (decaprenylphosphoryl-β-d-ribose 2-epimerase), is important for the synthesis of the cell wall of the mycobacterium, and the enzyme leucyl-tRNA synthetase (LeuRS) is necessary for protein synthesis.

**Figure 11:**
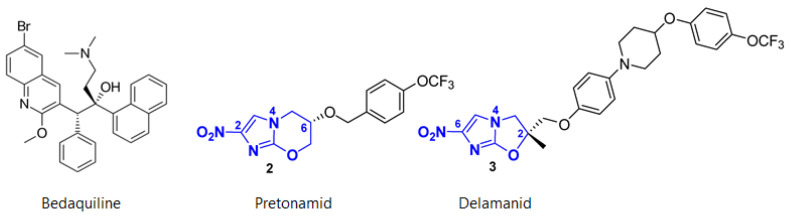
Bedaquiline, pretomanid, and dalamanid.

The most recently approved drugs for the treatment of multidrug-resistant pulmonary tuberculosis (MDR-TBC) are bedaquiline (approved in 2012) and delamanid (2014). Bedaquiline (marketed as Sirturo^®^) is, chemically, a diarylquinoline (Figure 11) [56]. This compound is an absolutely innovative drug, as it presents an unprecedented mechanism of action: It inhibits the ATPases proton pump that supplies ATP to the mycobacterium. Further data are needed to determine whether the benefits of bedaquiline outweigh its risks and, consequently, to define its role in the management of MDR-TB.

Nitroimidazoles are heterocyclic nitro-derivatives. In the 1990s, it was observed that metronidazole (5-nitroimidazole), belonging to this class, had moderate bactericidal activity against *M. tuberculosis* in anaerobic conditions. Subsequent studies led to the discovery of other nitroimidazoles, starting with the formula of metronidazole, which were more effective against mycobacteria. The 2-nitroimidazole replaced in positions 1 and 5 were the first nitroimidazolic compounds with antitubercular activity [57]. They are currently one of the most promising classes of antituberculosis agents in clinical research. Delamanid (Deltyba^®^, OPC-67683 in clinical development, Figure 11), approved by the FDA in 2014, is a 6-nitro-2,3-dihydro-imidazo-oxazole belonging to the class of nitroimidazoles and works by blocking the synthesis of the mycolic acids that make up the cell wall of *M. tuberculosis.* Delamanid has also been considered effective for the form XDR-TBC (extensively resistant), which is very difficult to treat and for which there are limited treatment options; it is common especially in India and southeast Asian countries. This is an important achievement. In August 2019, the FDA approved pretomanid (Dovprela^®^, PA-824 in clinical development, Figure 11), the first antitubercular bicyclic nitroimidazo-oxazine successfully developed and registered by TB Alliance, a non-profit organization founded in South Africa in 2000 [58]. The suffix “preto” comes from the city of Pretoria, South Africa, where the drug was developed. In 2020, the drug also received marketing approval from EMA, in a combination regimen with bedaquiline and linezolid (BPaL regimen), to be taken for only 6 months (a real revolution compared to existing therapies) for the treatment of XDR tuberculosis in adults and MDR tuberculosis that did not respond to other conventional antibiotics. This regimen was effective in 89% of the cases recorded in the clinical trial, which assessed the use of the same antibiotics in the MDR and XDR forms of tuberculosis. Moreover, it is also included in the new BPaMZ regimen, consisting of bedaquine, pretomanid, moxifloxacin, and pyrazinamide.

The mechanism of action is very complex. *Mycobacterium* can live in both aerobic conditions and hypoxia. Under aerobic conditions, the drug inhibits the biosynthesis of mycobacterium proteins and lipids; in particular, pretomanid blocks the transformation of hydroximicolic acid into ketomycolate (i.e., mycolic acids that, together with arabinogalattans and lipoarabinomannans, make up the wall of mycobacterium), with subsequent accumulation of hydroximicolic acid and depletion of ketomycolates [59].

Moreover, pretomanid also blocks the cellular respiratory processes of mycobacterium in an anaerobic environment through the release of nitric oxide, which kills *M. tuberculosis*. Thus, pretomanid is effective on both replication and latent *M. tuberculosis* cells, aerobically and anaerobically. The mechanism of action is therefore completely innovative. This was observed in laboratory experiments: Pretomanid-treated bacteria showed, in vitro, a different pattern of metabolites (especially with regard to the metabolic pathways of fatty acids, proteins, and the pentose-phosphate) than bacteria that received other antitubercular antibiotics [59]. The SAR of pretomanid shows that the enantiomer S is the most active; moreover, the presence of a nitro group in position 2 of the imidazole ring, the lipophilic tail in position 6 of the oxazinic ring, and the rigidity of the bicyclic system are crucial for antitubercular activity. These important portions are also found in other nitroimidazole antibiotics (CGI-1734 and TBA-354, in phase I clinical development).

Delamanid has notable affinities with pretomanid. Both delamanid and pretomanid are lipophilic, as required to penetrate the wall of the mycobacterium. Pretomanid is available in tablets for the treatment of pulmonary MDR and XDR tuberculosis; however, it is not active against extra-pulmonary tuberculosis, a particular form that fortunately represents only 5% of all existing TBC forms. Thanks to the latest drugs, the most aggressive and severe forms of tuberculosis resistant to traditional drugs are more treatable.

Tuberculosis is the infectious disease that has caused the greatest number of deaths ever, that’s why research has witnessed remarkable growth, also thanks to the growing investments and collaborations promoted and stimulated by the United Nations General Assembly and the TB Alliance.

## 6. Future Perspectives and Conclusions

Only two antibiotics of the eight approved since 2017 represent a new chemical scaffold [10]. The remaining antibiotics are actually derivatives of existing classes of compounds that bring benefits and advantages over traditional antibiotics.

The eight new antibiotics all have activities against ESBL (extended spectrum β-lactamase) enzymes; most of them are effective against carbapenem-resistant *Enterobacteria* (KPC producers), while very few compounds are active against carbapenem-resistant *P. aeruginosa* and multidrug-resistant *A. baumannii*. Unfortunately, there are still an extremely limited number of therapeutic alternatives for the latter. These antibiotics are mainly used in the treatment of cUTI and cIAI. Further scientific evidence is needed to assess their actual effectiveness in the treatment of other infections. Note that the combination of vaborbactam, meropenem, and plazomycin was included in the WHO Model List of Essential Medicines.

There is significant progress in research: The number of new effective antibiotics against Gram-negative bacteria has increased.

Most of the compounds approved and in clinical development from 2017 to today, whose targets are pathogens included in the list drawn up by the WHO in 2016 (critical priority, high, and medium), consist of combinations between a β-lactam and a β-lactamase inhibitor. Cefiderocol is the only antibiotic that is active against all three pathogens of critical priority, along with the compound called SPR-206 phase I (an analogue of polymyxins with an excellent antibacterial spectrum). At the end of 2020, there were 43 antibiotics in clinical development, of which, 15 were Phase I, 13 in Phase II, and 13 in Phase III. As many as 19 antibiotics are shown to be effective in vitro in the treatment of infections caused by pathogens of the so-called ESKAPE group, an acronym that includes the *Enterococcus faecium*, *S. aureus*, *K. pneumoniae*, *A. baumannii*, *P. aeruginosa*, and *Enterobacter* species, responsible for the six main nosocomial infections related to care [60].

It is, of course, essential that the new antibiotics developed do not have cross-resistance with other existing compounds. In fact, the search for new antibacterial drugs that result from the modification of traditional antibiotics is also based on knowledge of cross-resistance mechanisms. However, finding innovative chemical structures with new targets and binding sites is very difficult and yields fewer results than other approaches [61]. Furthermore, in addition to the small and large molecules that have been analyzed in this review, there are other potentially effective non-traditional approaches such as fecal bacteriotherapy (also called fecal microbiome transplantation) in the treatment of recurrent *C. difficile* infections. Other non-traditional approaches (such as immunomodulators and phage products) have not yet entered clinical development due to considerable obstacles. Unfortunately, unfavorable market trends remain: Although public investments in the development of new antibiotics have increased slightly in recent years (mainly from Germany, the United Kingdom, and the United States, thanks to organizations such as BARDA, CARB-X, and GARDP), private investment has fallen further. Many pharmaceutical companies are abandoning research in this area, not least because of the high costs involved in the clinical development of a new antibiotic. In view of the rather long time required for clinical development, 11 new antibiotics are expected to be approved in the next five years, while many compounds are likely to remain stagnant in Phases II and III due to the costs involved.

### 6.1. Has the SARS-CoV-2 Pandemic Affected Antibiotic Resistance?

A study conducted by the American non-profit organization The Pew Charitable Trusts, published in March 2021, examines the data of about 6000 SARS-CoV-2 positive and hospitalized patients in the United States, analyzing the period from February to July 2020 [62]. The data collected show that half of patients (52%) received antibiotic treatment in the first six months of the pandemic, increasing to 90% in March and April. On the other hand, 36% of hospital admissions led to the use of several antibiotics at the same time. Antibiotics were prescribed, in fact, to prevent bacterial infections secondary to viral infection, often even before the bacterial infection was confirmed. Another factor that contributed to worsening the situation was the difficulty, on the part of medical staff, in distinguishing bacterial pneumonia from SARS-CoV-2 viral pneumonia, evident especially in the first months of the emergency, when knowledge about SARS-CoV-2 was extremely limited.

The results suggest that, most likely, there was an excessive prescription of such drugs: Many patients, in fact, did not need antibiotic treatment. Bacterial infections can actually occur in patients diagnosed with viral infections, resulting in further deterioration of the clinical condition of the patient and complicating the therapy. The 20% of SARS-CoV-2 positive patients examined were affected by bacterial pneumonia, and in particular, community-acquired pneumonia. In 96% of the COVID-19 cases, antibiotics were administered within the first 48 h of admission to hospital. Few patients received other antibiotics in the period after 48 h after admission. However, considering patients who were given at least one antibiotic, only 33% had a confirmed diagnosis of community-acquired bacterial pneumonia. This means that in the remaining cases (67%), the antibiotic was prescribed unnecessarily, helping to fuel the phenomenon of antibiotic resistance.

Moreover, the study showed that the most frequently used antibiotics were macrolide azithromycin (used for more than 50% of hospital admissions), ceftriaxone (42%), vancomycin (25%), and the piperacillin-tazobactam association (23%). These antibiotics are commonly prescribed precisely for the treatment of bacterial pneumonia. Azithromycin has been administered in many subjects with interstitial pneumonia from SARS-CoV-2, as it is usually used to eradicate *Legionella* or *Chlamydia*, which can cause a similar pneumonia. It should be added that some patients (29%) have been treated with antibiotics that can increase the risk of contracting the pathogen *C. difficile*. The massive use of antibiotics during the pandemic, especially those with a broad antibacterial spectrum, risks hindering and slowing down the progress and results achieved by research in recent years. Some situations and particular factors can favor or prevent the transmission of MDR organisms: A study reported in the *Journal of Hospital Infection* from 2020 analyzes the potential impact of the SARS-CoV-2 pandemic on hospital transmission of these pathogens [63]. It is even more evident, given the current delicate situation, that the efforts of recent years will soon have to lead to the development of more and more antibiotics effective against multidrug-resistant organisms. However, it is not only antibiotics that are being cited: Recently, numerous research groups are focusing on new therapeutic approaches, which are one more weapon in the fight against antibiotic resistance.

### 6.2. Nanomedicine for Treatment of Infective Diseases

A possible strategy could be the destruction of the extracellular matrix that constitutes the bacterial biofilm (aggregations of microorganisms that form surface-adherent films). About 60% of microbial infections are associated with biofilm formation, as the bacteria organized in that structure are able to resist multiple antibiotics and the host’s immune system. The destruction of the biofilm leads to the release of bacteria that, therefore, regain sensitivity to the action of antibiotics. Research groups are currently studying polymeric lipid nanoparticles involving the conjugation of ramnolipids (biosurfactants secreted by the pathogen *P. aeruginosa*) and polymer nanoparticles in order to combat the resistance of *H. pylori* bacterial biofilm to commonly used antibiotics [64].

This system contains clarithromycin encapsulated in a polymeric core of chitosan; above all, it has antibacterial properties, also managing to prevent the formation of biofilm and bacterial adhesion. By the same principle, rhamnolipid-coated silver and iron oxide nanoparticles have been developed, which have been shown to be effective in eradicating *S. aureus* and *P. aeruginosa* biofilms [65].

Other structures that have been evaluated for their potential as release systems for antimicrobial drugs are crystalline liquid non-lamellar nanoparticles; they are made up of several amphiphilic structures with a large surface and are able to encapsulating both hydrophilic and hydrophobic drugs [66]. An example is the positively charged nanoparticles containing rifampicin, which showed lower MIC values respect to non-encapsulated rifampicin by inhibiting the growth of *S. aureus* [67]. There are also combinations between nanoparticles and natural compounds: Rodenak-Kladniew examined the incorporation of chitosan and eugenol (a natural phenolic compound) within a lipid matrix containing the antibiotic ofloxacin [68]. The results showed increased bactericidal action against *P. aeruginosa* and *S. aureus*.

Among the new systems for the release of antibiotics is the use of polymeric materials that respond to pH and the presence of enzymes at the site of infection, which allow the release of the active ingredient precisely where the infection occurs. Other approaches under study are antimicrobial oligonucleotides and photodynamic therapy [69]. A system has been developed that involves loading the antibiotic ciprofloxacin into photoactivable liposomes; the authors report that about 90% of the active substance was released in less than 30 s [70].

Combined therapy is often preferred to monotherapy to treat multidrug-resistant strains because the simultaneous use of several antibiotics with synergistic action allows, in fact, avoiding antibiotic resistance, increasing the antimicrobial spectrum, and decreasing the side effects of therapy. Co-encapsulation of several antibiotics into nanosystems can offer significant benefits: Research groups have manufactured liposomes with ciprofloxacin and colistine to treat *P. aeruginosa* infections [71]. In vitro results have shown that combined therapy is more effective than monotherapy.

Nano-antibiotics are another promising line of research. The transformation of therapeutic agents into corresponding structures at the order of the nanoscale can modify their chemical–physical properties, increase the bioavailability of the drug, and improve its interaction and penetration into the bacterial wall and therefore its effectiveness against resistant strains. Clarithromycin formulations in nanocrystals have shown activity against multidrug-resistant *H. pylori*: Nanocrystals allow the drug to be directed to the desired site with a better therapeutic profile than clarithromycin suspension and powder [72]. Some nanostructured systems containing antibiotics and antimicrobial peptides are currently in clinical trials [69]. For example, numerous inhalation formulations of liposomal ciprofloxacin are in Phase I, II, and III, while a formulation of liposomal amikacin for the treatment of recurrent *P. aeruginosa* infections in patients with cystic fibrosis is already in Phase III of clinical development.

In nanomedicine, the advantages of using liposomes as antibiotic carriers range from the reduction of toxicity to the improvement of pharmacokinetic parameters and in particular, of biodistribution. The fusion of liposomal vesicles with the external membrane of the bacterium allows a better release of the antibiotic and a better penetration into the bacterial cell. Although nanostructured systems are more traditionally used in oncology and cancer immunotherapy, they could also represent a revolution in antibiotic delivery, where much remains to be discovered. It is clear that translating such antibiotic-loaded nanostructured systems into clinical practice requires further investigation and efforts to combat antibiotic resistance, which today is something of a “silent” pandemic. Thanks to the use of nanoparticles, it will be possible to overcome the resistance mechanisms: These structures allow a better internalization of antibiotics, both hydrophilic and hydrophobic, that are not enzymatically inactivated and selectively reach the site of infection. As reported in numerous studies, the use of nanoparticles loaded with antibiotics or antimicrobial peptides shows significant reductions in MIC values compared to the corresponding values expected from the use of non-encapsulated active ingredients. In this way, it is possible to inhibit the development of antibiotic resistance mechanisms. Despite the promising results obtained in vitro, there are still few formulations included in clinical trials, also due to the high costs of these preparations.

In 2014, Jim O’Neill published an article commissioned by the British government entitled “*Antimicrobial Resistance: Tackling a crisis for the health and wealth of nations*”, in which there was a completely catastrophic projection: The author estimated, in fact, that by 2050, there will be approximately 10 million deaths per year caused by antibiotic resistance, even higher than the sum of deaths from cancer and diabetes [73]. This prediction has also been mentioned in numerous other publications, including the media and health authorities. To achieve that tragic value, the model used in the report multiplied the number of bloodstream infections nationwide by national resistance rates, as reported by the European Antimicrobial Resistance Surveillance Network. These are, of course, projections on a very sensitive subject, so it is still difficult to express an accurate opinion. Solid data on antibiotic resistance is needed, not only in Europe, as examined in the report, but also in less developed countries, to take concrete action.

The progress of research in the coming years will be crucial, and the drugs analyzed in this review represent only the beginning, but they are a significant step forward which, combined with individual behavior and human responsibility, can really make a difference and allow for an inversion of current estimates.

## Data Availability

The data presented in this study are openly available in a publicly accessible repository.
